# Clinical efficacy and safety outcomes of anlotinib therapy in sarcoma: a systematic review and meta-analysis

**DOI:** 10.1186/s43046-026-00384-5

**Published:** 2026-07-24

**Authors:** Hasan Matar, Ahmad Melhem, Abdallah Shawwa, Taleen Yousef, Mohammad Alananbeh, Enad Alsalim, Eman Al-refai, Malaak Abuhwaij, Dina Elayan

**Affiliations:** 1 Jordan University of Science and Technology, Irbid, Jordan; 2 University of Jordan, Amman, Jordan; 3 Yarmouk University, Irbid, Jordan

**Keywords:** Anlotinib, Sarcoma, Tyrosine kinase inhibitor, Progression-free survival, Overall survival, Meta-analysis

## Abstract

**Purpose:**

Sarcomas are rare, aggressive and unpredictable tumors that arise from mesenchymal tissues. Despite treatment, outcomes for advanced or metastatic cases remain poor. Anlotinib is a new oral tyrosine kinase inhibitor that blocks multiple angiogenic pathways and has shown encouraging results in solid tumors. This review aims to summarize and clarify the current evidence on anlotinib’s role in treating sarcoma.

**Methods:**

A systematic search across five major databases up to February 2025 identified clinical studies that evaluate anlotinib in sarcoma patients. Eligible studies included randomized controlled trials and observational studies evaluating anlotinib in advanced or metastatic sarcoma. Pooled estimates for median progression-free survival (mPFS) and overall survival (mOS) were calculated using random-effects models.

**Results:**

Twenty-one studies involving 1,230 patients were included. The pooled mPFS was 6.7 months and the mOS was 19.3 months. Results varied widely due to differences in sarcoma subtype, disease stage, and prior therapies, yet most studies showed meaningful tumor control and manageable toxicity.

**Conclusions:**

Anlotinib appears to be a promising therapy for refractory or metastatic sarcomas. It offers modest yet real improvements in survival and quality of life. Larger, multicenter and biomarker-guided studies are needed to define which patients benefit most and how this drug can be best integrated into future treatment strategies.

## Introduction

Sarcomas are rare, heterogeneous mesenchymal tumors that tend to develop in any of the body’s tissues and organ, of which more than 70 forms of sarcoma have been classified while molecular profiling advancements will most probably increase their quantity over the upcoming years [1]. Globally, sarcoma prevalence is approximately 5 per 100,000 persons per year, thus qualifying it as a rare tumor by international standards [2].

In 2021, it was estimated that there were over 480,000 new sarcoma diagnoses and over 50,000 deaths around the world, equivalent to 1.16 age-standardized incidence per 100,000 person-years and mortality of 20.54 per 100,000 person-years. The sarcoma burden rose globally by approximately 42.8% during the past two decades due to population growth and aging, ongoing chronic disease, and shifting environmental and social risk determinants. Even with increasing burden, sarcomas are under-investigated in drug development. Between 2000 and 2024, only a few drugs were approved by regulatory bodies like the FDA, NMPA, PMDA, and EMA for sarcoma treatment in general, and most of them were approved for the overall indications of sarcoma but not for any subtype [3].

Clinical response in metastatic or recurrent soft tissue and bone sarcoma patients continues to be poor. Nearly 50% of the patients either present initially with de novo metastasis or later develop recurrence, and most of the patients are also resistant to standard chemotherapy [4–7]. Consequently, there is a critical need for the discovery and research of more effective therapeutic strategies.

Anlotinib is an oral multi-targeting tyrosine kinase inhibitor (TKI), a novel drug with therapeutic potential for treatment of a wide array of solid neoplasms [8–12]. It was first approved in 2018 for refractory NSCLC following two or more previous systemic therapies [8]. Anlotinib has also been shown to have exceptional antitumor efficacy on a wide array of other neoplasms, such as ovarian, renal, and lung cancer [9, 10]. Anlotinib exerts its action through selective inhibition of VEGFR-2 and − 3, FGFRs 1–4, PDGFRα/β, c-Kit, Ret, DDR1, and other tumor microenvironment, angiogenesis, cell proliferation-associated kinases [11, 12]. With pharmacologic favorability and with very minimal drug–drug interaction, it has been tried even with cytotoxic drugs, and good progression-free and overall survival outcomes have been achieved with first-stage trials [10].

In phase II preclinical and clinical trials, anlotinib has also achieved success in refractory metastatic soft tissue sarcomas [12] and by all accounts would be an agent to use to infuse in such refractory patients [4]. To date, however, there still is no data to definitively confirm its position in the treatment of sarcoma. Our systematic review here will synthesize up-to-date clinical evidence of anlotinib use in sarcoma treatment and shed light on its future use, improve patient outcomes, and guide future treatment. By putting study results mentioned within our systematic review under the limelight, we aim to close this core gap in evidence and explore its effects on practice.

## Methods

This systematic review of meta-analysis followed the Preferred Reporting Items for Systematic Reviews and Meta-Analyses (PRISMA) guidelines [13]. As the present paper is based entirely on previously published studies, no ethical approval was required.

### Search strategy and data sources

A systematic search approach was conducted across five databases: PubMed, Web of Science, Scopus, Embase and Cochrane from the inception date until February 2025, with no restrictions imposed on language, publication date, study population or study type. The search strategy employed the medical subject headings (MeSH) terms identified via PubMed. The phrases ‘Anlotinib’, ‘Sarcoma’, ‘osteosarcoma’ and ‘liposarcoma’ were used and combined using Boolean operators (AND, OR). The protocol was adapted and applied to the remaining selected databases to ensure comprehensive coverage.

### Eligibility criteria

Studies were included based on predefined exclusion and inclusion criteria. The inclusion criteria were (1) observational studies (cohorts or case control) or Randomized control trials (RCTs) (2) all studies evaluating the efficacy of anlotinib in patients with metastatic or advanced localized sarcoma (3) patients treated with anlotinib alone or combined with chemotherapy, radiotherapy and surgery (4) studies reporting efficacy outcome. Case reports, reviews, comments, letters, animal studies and studies reporting anlotinib combined with immunotherapy were excluded.

### Study selection

The identified studies were imported into EndNote for detection and exclusion of duplicates. Remaining articles were screened by title and abstract using Rayyan, an AI assisted tool for systematic review screening. Full-text articles of potentially relevant studies were then retrieved and assessed in detail based on the predetermined inclusion and exclusion criteria. Two authors independently performed the screening process, blinded to each other’s results. Disagreements were resolved by third author through discussion.

### Data extraction

A total of 21 studies that met our eligibility criteria were extracted based on predefined variables of interest into an Excel spreadsheet. The first sheet encompassed aspects of the author’s names, country of origin, type of study, type of sarcoma, number of patients, gender, mean age, ECOG scores, study objectives and conclusions. The following other sheets were created for a complete extraction of relevant information: eligibility criteria, sarcoma subtypes, prior treatment and history metastasis. All data was extracted by two researchers independently into the standardized data extraction forms. Discrepancies were resolved by consensus.

### Risk of bias assessment

We used Cochrane Risk of Bias 2 (ROB2) tool to assess the internal validity for all randomized control studies (RCTs) included, which is structured into a fixed set of five domains of potential bias: randomization process, deviations from intended interventions, missing outcome data, outcome measurement and selection of reported results. Each domain contains a series of ‘signalling’ questions (approximately 13–14 in total). Responses to these questions were used to determine overall risk of bias, categorized as ‘low risk of bias’, ‘some concerns’ or ‘high risk of bias’. For the rest of the studies, the National Institutes of Health (NIH) was used, this quality assessment tool was based on a scoring system, studies between (1–6) were referred to as ‘poor’, (7–8) is considered ‘fair’ and (9–14) is classified as ‘good’. Judgments were made independently by two reviewers; disagreements were resolved by discussion between reviewers.

### Statistical analysis

Statistical analyses were performed using R software version 4.1.2 with the metafor package version 4.8-0 [14]. Continuous outcomes were expressed median with corresponding 95% confidence intervals (CI), calculated using the generic inverse variance method. A random-effects model was applied to account for anticipated heterogeneity across studies. Statistical heterogeneity was evaluated using Cochran’s Q test and the I² statistic, with I² values above 50% indicating moderate heterogeneity and values above 90% indicating substantial heterogeneity. A p-value of less than 0.05 was considered statistically significant. Publication bias was assessed visually using a funnel plot, where study effect sizes were plotted against their standard errors. In the absence of bias, the plot is expected to form a symmetrical inverted funnel. Egger’s regression test was additionally applied to statistically evaluate funnel plot asymmetry.

## Results

### Literature search results

**Fig. 1:**
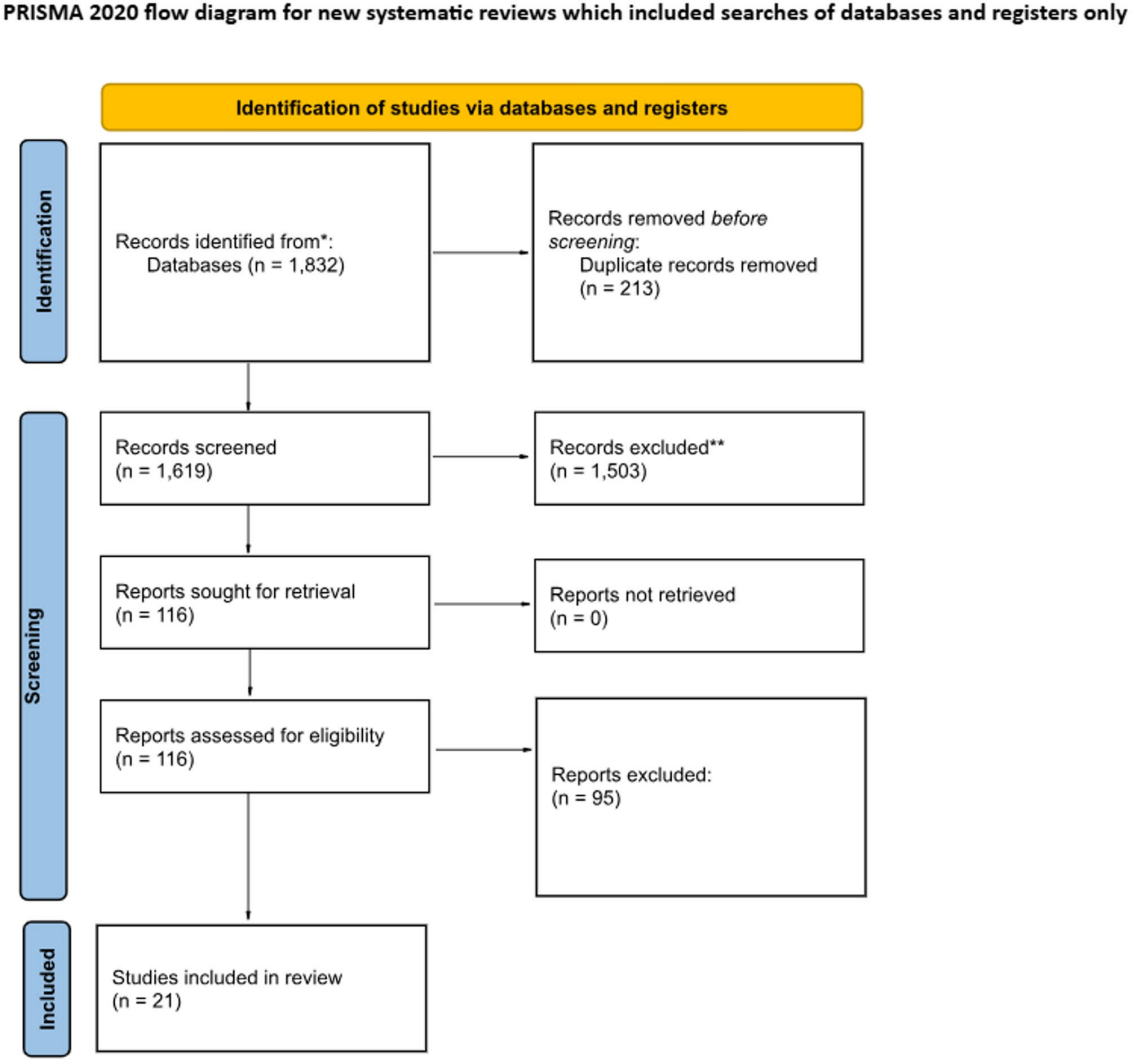
PRISMA flow diagram for systematic reviews summarizing the literature search process. Source: [13]. This work is licensed under CC BY 4.0. To view a copy of this license, visit https://creativecommons.org/licenses/by/4.0/

The systematic literature search yielded a total of 1,832 records. Following the removal of 213 duplicate entries, 1,619 unique studies remained for screening. After evaluation of titles and abstracts, 116 studies were deemed potentially eligible and retrieved for full-text assessment, all of which were accessible. Upon comprehensive full-text review, 21 studies fulfilled the predefined inclusion criteria and were subsequently incorporated into the final analysis. Fig. 1.

### Characteristics of the included studies

Our review included 21 studies published between 2020 and 2025 involving 1230 patients. Sixteen of these included studies were retrospective cohort studies and 5 RCTs [4, 12, 15–33]. The studies included different types of sarcomas as 4 studies included bone and soft-tissue sarcomas while 11 included only soft tissue sarcomas in general, 1 study included leiomyosarcomas, another included liposarcomas and another one included ASPS specifically, 2 studies only included bone sarcomas in general and 1 study included osteosarcomas specifically. All studies were conducted in China. The sample sizes of the included studies were low, ranging from 15 to 209 patients. The sex distribution of the included studies did not follow a specific pattern, as some studies included more males and other studies included more females. Most of the included studies had similar mean ages, approximately 46 years, while some studies included patients in different age groups. The ECOG status scores demonstrated that most patients had ECOG performance status of 0–1 (patients were fully active or only mildly limited), while a small subset of patients were classified as 2 (patients were ambulatory, self-care only and unable to work). Finally, the follow-up durations ranged from 8 to 46 months. Table 1.

**Table 1:** Characteristics of the included studies

Study ID	NCT NO.	Type of the study	Country	Type of sarcoma	Number of patients			Gender (M/F)	Mean age		ECOG status scores			Follow-up duration (months)
					Total	Anlotinib	Comparison		Anlotinib	Comparison	0	1	≥ 2	
Pang et al., 2025 [21]		Retrospective institutional	China	Bone and Soft-tissue Sarcomas	92	Anlotinib + Chemotherapy (47)	Chemotherapy (45)	59/33	41	38	84		8	46
Cai et al., 2022 [4]		Retrospective cohort	China	Bone and Soft-tissue Sarcomas	44	Anlotinib (23)	Anlotinib + Chemotherapy (19) Anlotinib + Radiotherapy (2)	21/23	48.5		30		14	13.6
Yao et al., 2022 [30]		Retrospective cohort	China	Bone and Soft-tissue Sarcomas	22	Anlotinib (9)	Anlotinib + Chemotherapy (9) Anlotinib + immunotherapy (4)	14/8	44.82					CR (13.50) PFS (16.50)
Zou et al., 2022 [18]		Retrospective cohort	China	Leiomyosarcoma	19	Anlotinib (12)	Anlotinib + Chemotherapy (2) Anlotinib + Radiotherapy (2) Anlotinib + immunotherapy (2) Anlotinib + immunotherapy + Chemotherapy (1)	5/14/.	52		5	5	9	15.85
Wang et al., 2020 [29]	NCT03951571	RCT	China	Soft tissue sarcomas	88	Anlotinib (44)	Placebo (44)	51/37	56.5	57.5	Placebo (25) Anlotinib (27)	Placebo (19) Anlotinib (17)	0	Anlotinib (34.13) Placebo (29.21)
Zheng et al., 2024 [28]		Retrospective cohort	China	Soft tissue sarcomas	94	Anlotinib (49)	gemcitabine-based chemotherapy (45)	36/58	50	44	Anlotinib (43) gemcitabine-based chemotherapy (38)		Anlotinib (6) gemcitabine-based chemotherapy (7)	17.7
Liu et al., 2020 [16]		Retrospective cohort	China	ASPS	47	Anlotinib (16)	pazopanib (31)	17/30	27.8		Anlotinib (11) pazopanib (19)	Anlotinib (5) pazopanib (12)	0	Anlotinib (36.8) pazopanib (35.6)
Ni et al., 2024 [26]		Retrospective cohort	China	Soft tissue sarcomas	14	Anlotinib + Chemotherapy (14)		9/5.	52.86					
Wang et al., 2020 [5]		Retrospective cohort	China	Soft tissue sarcomas	32	Anlotinib + Chemotherapy followed by Anlotinib maintenance therapy (32)		17/15	45		14	17	1	14
Li et al., 2024 [23]	NCT03792542	RCT	China	Soft tissue sarcomas	40	Anlotinib (40)		24/16	57.5		8	29	3	OS (36.04) PFS (12.81)
Zhang et al., 2022 [25]		Retrospective cohort	China	Soft tissue sarcomas	35	Anlotinib (30) Anlotinib + Chemotherapy (2) Anlotinib + Immunotherapy (2) Anlotinib chemoradiotherapy (1)		16/19	65		32		3	13.3
Tian et al., 2020 [24]		Retrospective cohort	China	Bone and Soft-tissue Sarcomas	110	Anlotinib (42) : OS (13) STS (29)	Apatinib (68): OS (19) ST (49)	OS (15/17) STS (42/36)	Anlotinib + OS (20.46) Anlotinib + STS (41.86)	Apatinib + OS (22.42) Apatinib + STS (41.10)	Apatinib + OS (11) Apatinib + STS (24) Anlotinib + OS (7) Anlotinib + STS (15)	Apatinib + OS (8) Apatinib + STS (25) Anlotinib + OS (6) Anlotinib + STS (14)	0	
Liu et al., 2021 [17]		Retrospective cohort	China	Bone sarcomas	48	Anlotinib (48)		29/19	24		26	13	9	8
Li et al., 2021 [32]		Retrospective cohort	China	Liposarcoma	17	Anlotinib (17)		9/8.	57.25					8.82
Zhang et al., 2022 [22]		Retrospective cohort	China	Soft tissue sarcomas	209	Anlotinib (112)	Combination therapy (62) Switch maintenance therapy (35)	106/103	48		170		39	18.7
Xu et al., 2023 [19]	NCT03890068	RCT	China	Soft tissue sarcomas	49	Anlotinib (49)		17/32	49.33		15	33	1	17.77
Li et al., 2021 [32]		Retrospective cohort	China	Osteosarcoma	15	Anlotinib (15)		9/6.	15.2					10.7
Tang et al., 2022 [31]	NCT03527888	RCT	China	Bone sarcomas	42	Anlotinib (42)		25/17	27.33		3	34	5	9.6
Liu et al., 2020 [16]		Retrospective cohort	China	Soft tissue sarcomas	21	Anlotinib (21)		12/9.	45.5					15.1
Chi et al., 2018 [12]	NCT01878448	RCT	China	Soft tissue sarcomas	166	Anlotinib (166)		100/66	45.5		50	96	20	45.5
Liu et al., 2021 [20]		Retrospective cohort	China	Soft tissue sarcomas	26	Anlotinib (14)	apatinib (7) pazopanib (4) lenvatinib (1)	17/9	34		18		8	26.9

### Quality assessment of the included studies

Twenty studies were evaluated using the National Institutes of Health (NIH) Quality Assessment Tool. Among these, all were judged to be of good methodological quality, demonstrating adequate study design, clear objectives, reliable outcome measures and appropriate statistical analyses. Only Li et al. 2020 showed methodological limitations that placed it at fair quality. The remaining study was evaluated using the ROB-2 tool and it was determined to be at high risk of bias due to issues related to selection of the reported results. Overall, the evidence base was considered robust, with the majority of studies showing low risk of bias and sound methodological rigor.

### Outcomes

#### Median progression-free survival (mPFS)

**Fig. 2:**
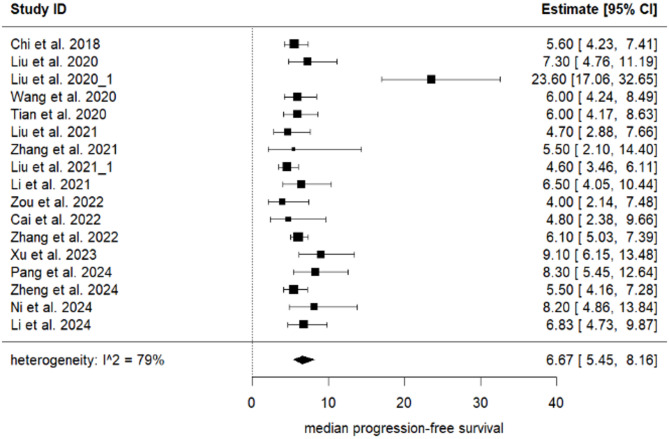
Pooled overall mPFS for anlotinib

Our analysis of 17 studies demonstrated that the pooled overall mPFS for anlotinib was 6.67 months (Fig. 2: Estimate: 6.67 months, 95% CI [5.45; 8.16], *P* < 0.0001). Very high heterogeneity was detected across the studies (tau^2^: 0.13, I^2^: 79.39%). The sensitivity analysis demonstrated that the exclusion of Liu et al. 2020_1 significantly reduced the heterogeneity detected and slightly reduced the estimate (estimate: 6.03 months, I^2^: 3.11%).

**Fig. 3:**
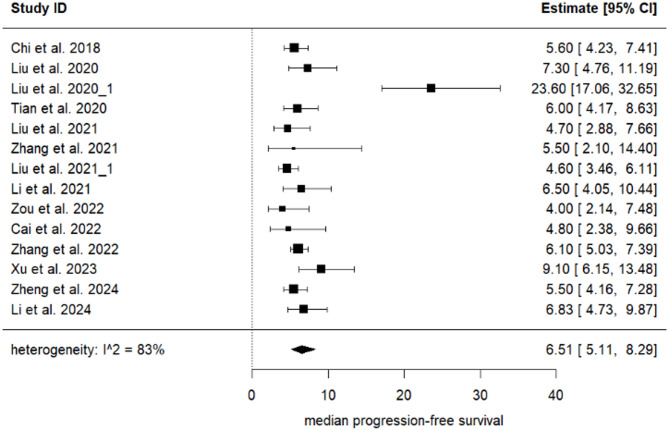
Pooled overall mPFS for anlotinib as a monotherapy

The analysis of 14 studies that used anlotinib as a monotherapy demonstrated that the pooled overall mPFS was 6.51 months (Fig. 3: Estimate: 6.51 months, 95% CI [5.11; 8.29], *P* < 0.0001). Very high heterogeneity was detected across the studies (tau^2^: 0.16, I^2^: 83.14%). The sensitivity analysis demonstrated that the exclusion of Liu et al. 2020_1 significantly reduced the heterogeneity detected and slightly reduced the estimate (estimate: 5.87 months, I^2^: 0.54%).

**Fig. 4:**
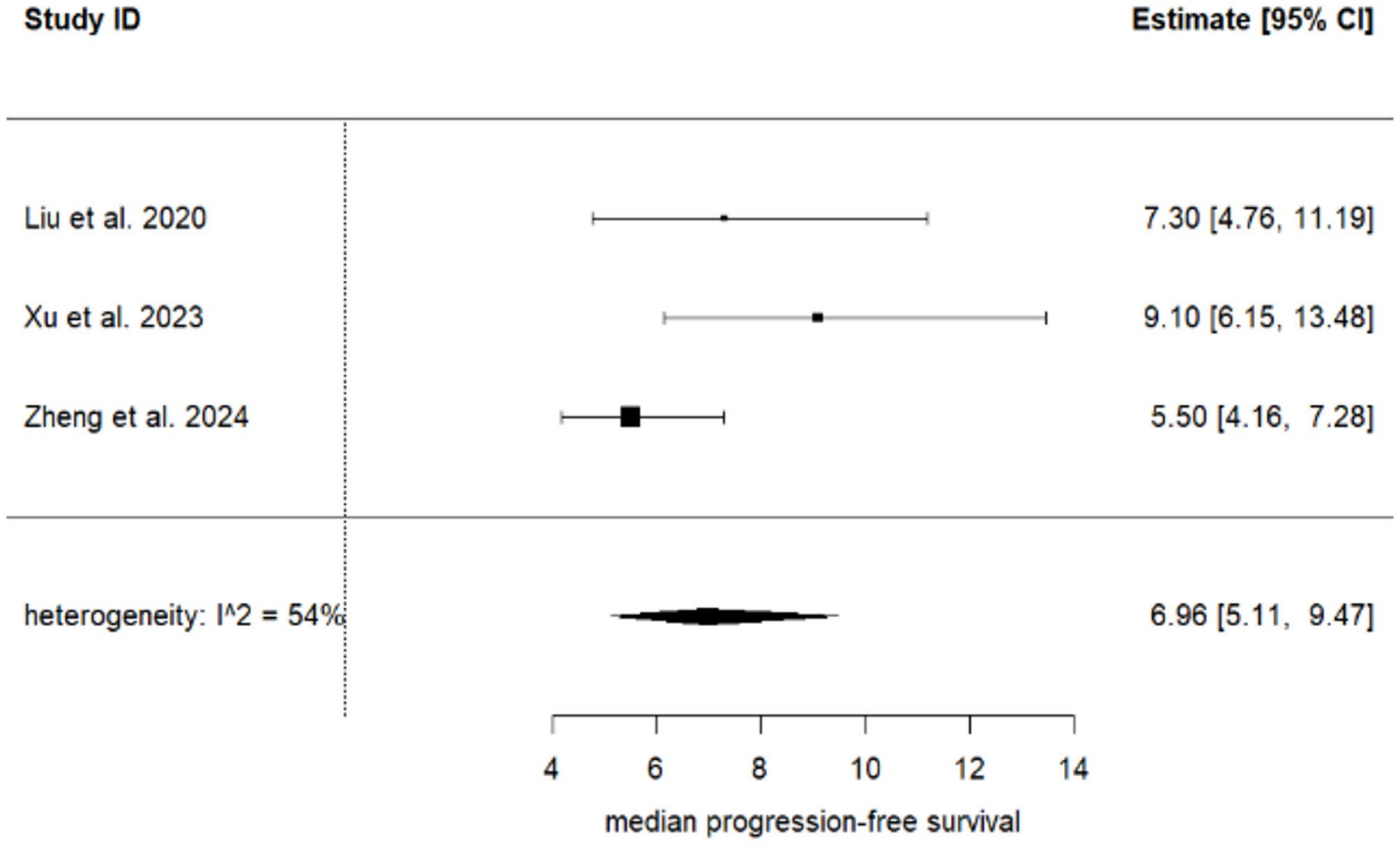
Pooled overall mPFS for anlotinib after chemotherapy failure

The analysis of 3 studies that used anlotinib after chemotherapy failure demonstrated that the pooled overall mPFS was 6.96 months (Fig. 4: Estimate: 6.96 months, 95% CI [5.11; 9.47], *P* < 0.0001). Moderate-high heterogeneity was detected across the studies (tau^2^: 0.04, I^2^: 54.03%).

**Fig. 5:**
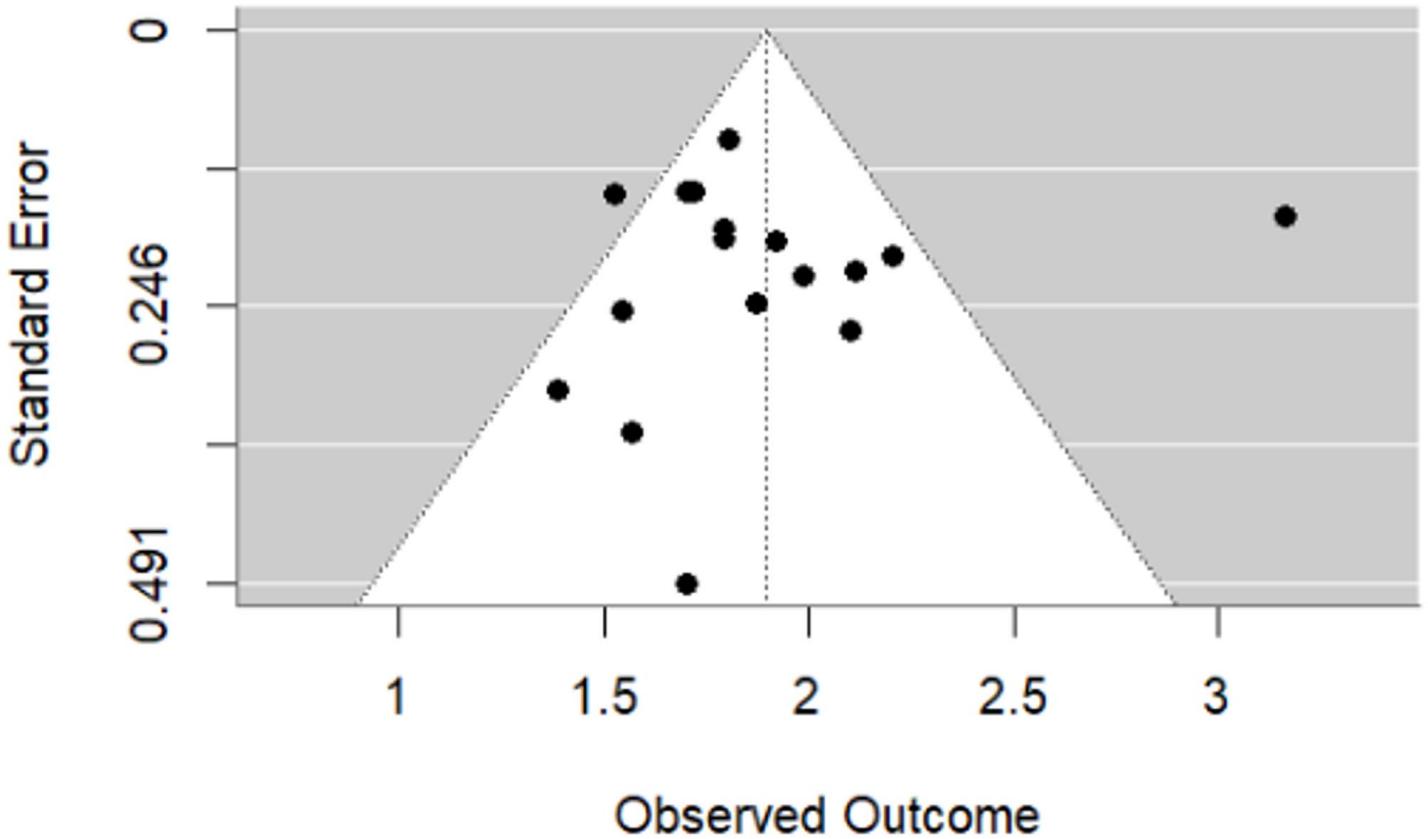
Funnel plot assessing potential publication bias in the mPFS for anlotinib

**Fig. 6:**
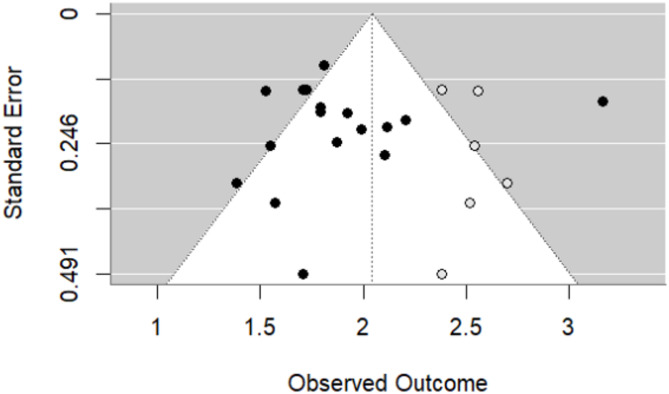
Adjusted funnel plot following the Trim and Fill imputation method for publication bias in the mPFS for anlotinib

The Egger’s test demonstrated that no significant publication bias was detected (*P* = 0.49). But the visual inspection of the funnel plot showed some asymmetrical distribution of the included studies. Consistently, the trim-and-fill method demonstrated that 6 studies were missing from the right side of the funnel plot, but they did not influence the results badly as they increased the estimate (estimate: 7.71 months, I^2^: 80.79%). Figs. 5 and 6.

#### Median overall survival (mOS)

**Fig. 7:**
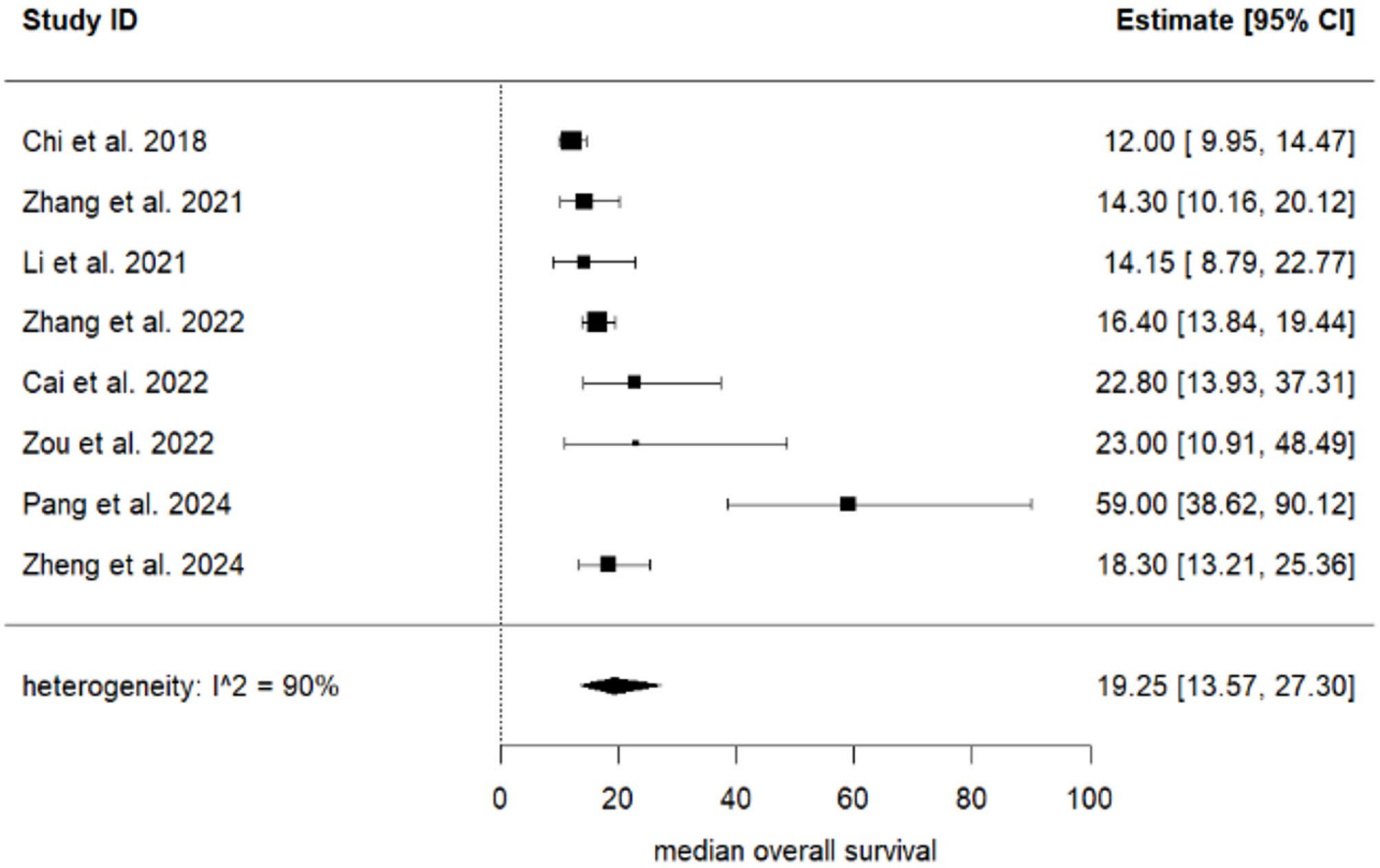
Pooled overall mOS for anlotinib

Our analysis of 8 studies demonstrated that the pooled mOS for anlotinib was 19.25 months (Fig. 7: 19.25 months 95% CI [13.57; 27.3], *P* < 0.0001). Very high heterogeneity was detected across the studies (tau^2^: 0.21, I^2^: 89.56%). The sensitivity analysis demonstrated that the exclusion of Pang et al. 2024 significantly reduced the heterogeneity detected and slightly reduced the estimate (estimate: 15.66 months, I^2^: 50%).

#### Safety

Safety results were reported heterogeneously across papers; thus, no pooled safety assessment could be conducted. In terms of the occurrence of side effects, most toxicities were grade 1–2 events including hypertension, hand-foot syndrome, fatigue, diarrhea, proteinuria, anorexia, mouth ulcers, lipids disorders, thyroid dysfunction, and elevation of liver enzymes [12, 15, 17–19, 22, 23, 27, 29]. The main adverse events in the refractory STS study were hypertension, high triglycerides level, and pneumothorax, recorded at the rate of 4.8%, 3.6%, and 2.4% of cases, respectively [12]. Grade 3–4 hypertension and hand-foot syndrome happened in 10% and 6% of patients, respectively, while in a switch-maintenance trial conducted by a single center, grade 3/4 adverse events developed in 28.6% of patients [17, 19]. Severe toxicities were observed at a higher rate among elderly patients with STS, when 37.1% of patients experienced grade 3/4 adverse events [22]. Higher toxicity rates were also observed in bone sarcomas with 54.8% of grade ≥ 3 adverse events in the recurrent/metastatic primary malignant bone tumor case and grade 3 adverse events in 33% of patients in metastatic osteosarcoma trials [15, 31]. In studies assessing the combination with chemotherapy, hematologic toxicity was more common with grade 3/4 leukopenia occurring in 19%, febrile neutropenia in 9%, anemia in 6%, thrombocytopenia in 3%, and hypertension in 6% of cases) [29]. Patients in the switch-maintenance study and post-ALTER0203 population had dose reduction in 23.8% and 14.8% of cases, while treatment discontinuation due to toxicity took place in 9.5% and 12.0% of patients [17, 25]. No deaths attributable to therapy were recorded in papers mentioning it as an adverse event [4, 15, 17, 25–27, 29, 31].

## Discussion

This systematic review and meta-analysis synthesizes the most recent clinical evidence on the efficacy and safety of anlotinib in patients with sarcomas. By pooling data from diverse study designs and sarcoma subtypes, it provides a more precise and comprehensive estimate of treatment outcomes. The pooled analysis increases statistical power, reduces random error and allows for a clearer understanding of anlotinib’s therapeutic role across heterogeneous sarcoma populations.

Sarcomas are rare malignant tumors of mesenchymal origin which account for about 1% of adult cancers and comprising more than 50 histological subtypes. Despite multimodal therapy, the prognosis for advanced or metastatic cases remains poor, with five-year survival rates below 20%. Anlotinib, a multitarget tyrosine kinase inhibitor of VEGFR, PDGFR, FGFR and c-Kit, has shown promising efficacy and tolerable safety in refractory or metastatic sarcomas [15].

Our analysis demonstrated that the pooled overall mPFS for anlotinib was 6.67 months with a high heterogeneity across studies (Estimate: 6.67 months, 95% CI [5.45; 8.16], I^2^: 79%). The high heterogeneity detected can be attributed to several methodological and biological factors. The included studies involved highly diverse histologic subtypes, this heterogeneity strongly influences responsiveness and consequently the mPFS outcomes [24]. Also, anlotinib was used in diverse contexts as monotherapy, combination therapy and varying treatment lines. Finally, discrepancies in baseline characteristics, dosing schedules and follow-up durations contributed to inconsistent survival estimates too. The mPFS ranged from 4 to 23.6 months, all studies reported close mPFS except for Liu et al. 2020_1 which reported a significantly higher mPFS which is due to the inclusion of patients who had already shown good tolerance and disease control with prior TKI therapy. This selective inclusion of clinically stable patients led to an overestimation of anlotinib’s mPFS. The analysis of studies that used anlotinib as a monotherapy demonstrated a similar pooled overall mPFS for anlotinib with a very high heterogeneity, so giving anlotinib as a monotherapy or combination did not explain the heterogeneity. Also, the analysis of studies that used anlotinib after chemotherapy failure demonstrated similar results too, with Moderate-high heterogeneity and a wider confidence interval related to the low number of studies included in the analysis.

Our analysis demonstrated that the pooled overall mOS for anlotinib was 19.25 months with a high heterogeneity across studies (Estimate: 19.25 months, 95% CI [13.57; 27.3], I^2^: 90%). The high heterogeneity detected can be attributed to the same reasons explaining the heterogeneity of mPFS. The mOS ranged from 12 to 59 months, all studies reported close mOS except for Pang et al. 2024 which reported a significantly higher mOS which is due to the inclusion of patients with favorable characteristics. Most patients had excellent performance status (ECOG 0–1) and anlotinib was used as first-line therapy in combination with chemotherapy. The cohort also included histologic subtypes known for slower progression and better TKI response like leiomyosarcoma and ASPS and the long median follow-up of 46 months with several patients still alive made the survival estimate statistically immature.

Heterogeneity in this meta-analysis arises from the fact that the use of anlotinib was explored across three different clinical settings: refractory or progressive disease after initial systemic treatment, maintenance therapy after chemotherapeutic disease control, and combination therapy using chemotherapeutic agents [12, 17, 19, 21, 26, 29]. These three different clinical settings cannot be considered comparable, refractory cohorts included patients with progressing and resistant tumors, maintenance cohorts included patients who were able to achieve stable disease or partial response following their chemotherapy, thus constituting a patient subset with a better prognosis [12, 17, 19]. It is crucial to note that both the single center-switch maintenance study and the prospective trial ALTER-S006 require their patients to undergo disease control after undergoing chemotherapy before being enrolled into the anlotinib arm, meaning that the patient subset is expected to have better tumor biology compared to other subsets where patients are enrolled at the time of active disease progression [17, 19]. Accordingly, the pooled estimation of the effect on mPFS obtained from these trials together with studies evaluating refractory disease may be shifted towards a higher value due to this heterogeneity, leading to an overestimation of the drug’s efficacy for patients with actively progressing tumors [12, 17, 19, 25]. Combination therapy represents yet another heterogeneity factor as a good outcome may be attributed to chemotherapy, anlotinib, or both [4, 21, 26, 29]. An illustration for this problem can be given by the real-world post-ALTER0203 study which specifically classified patients based on their treatment modality (monotherapy, combination or switch maintenance) and found that treatment pattern was associated with both PFS and OS of patients [25]. Thus, when considering the efficacy estimates in this meta-analysis, it is vital to consider that the observed benefits are results of using the drug under different circumstances and cannot be extrapolated into a universal efficacy value for all patients with advanced sarcomas [12, 17, 19, 25]. This problem brings us to a wider dilemma within the field of advanced sarcoma management - the optimal positioning of targeted angiogenesis inhibitors, whether it be as salvage monotherapy, as a maintenance treatment, or in combination with chemotherapy depending on specific subtype [12, 17, 19, 21, 25, 26, 29]. While anlotinib maintenance therapy appears to be promising, showing 9.1 months’ median PFS and a 94% disease control rate in the setting of ALTER-S006, it is important to emphasize that patients enrolled in the trial had been previously benefiting from their initial chemotherapy [19]. Such reasoning fits well into the larger picture of angiogenesis inhibitor maintenance therapy as exemplified by the EREMISS randomized phase II trial which evaluated regorafenib maintenance treatment after first-line doxorubicin-based chemotherapy and demonstrated delayed disease progression compared to placebo in advanced non-adipocytic soft tissue sarcoma [34].

A notable strength of this meta-analysis is that all included studies were conducted in Chinese populations. This provides a unique insight into the efficacy of tyrosine kinase inhibitors in an exclusively Asian cohort, where pharmacokinetic and pharmacogenomic differences may influence drug response. Such population-specific evidence has not been extensively reported for other TKIs in sarcoma and represents an important contribution to literature.

Pazopanib is an oral TKI that has multiple targets which makes it mechanistically similar to anlotinib in suppressing angiogenesis. In the PALETTE phase III trial, pazopanib significantly improved mPFS to 4.6 months in pretreated soft-tissue sarcoma, but the mOS difference was not statistically significant compared to placebo. Real world data confirmed durable disease control and manageable toxicity, positioning pazopanib as the benchmark anti-angiogenic comparator for newer TKIs like anlotinib [35]. A previous meta-analysis on soft-tissue sarcoma reported that pazopanib achieved a pooled mOS of 10.3 months and a mPFS of 5.3 months in previously treated advanced sarcoma which confirms its role as a second-line treatment [36].

Regorafenib is another oral TKI that shares a broad anti-angiogenic spectrum with anlotinib. In the REGOSARC phase II trial, regorafenib prolonged mOS to 3.7 months in leiomyosarcoma and 5.6 months in synovial sarcoma compared with roughly 1 month on placebo. Despite higher incidences of hand foot syndrome and hypertension, regorafenib demonstrated consistent disease stabilization in refractory sarcomas, reinforcing the clinical relevance of VEGFR/FGFR blockade shared by anlotinib [37]. Comparisons of anlotinib with pazopanib and regorafenib should be interpreted cautiously, as cross-trial comparisons are limited by differences in histology, treatment line, patient selection, and study design.

Specifically, several of the included cohorts have particular clinical relevance to pediatric and adolescent/young adult (AYA) populations due to inclusion of younger patients in the bone sarcoma studies: an unresectable/metastatic bone sarcoma cohort with a median age of 24 years, a recurrent/metastatic primary malignant bone tumor trial recruiting patients from 14 years of age, and a metastatic osteosarcoma cohort recruiting patients aged 10–21 years with a mean age of 15.2 years [15, 20, 31]. Moreover, ASPS also merits special attention regarding the AYA population since this malignancy predominantly affects adolescents and young adults, while the included ASPS study specifically notes that patients with ASPS require longer treatment with TKIs due to increased sensitivity to anti-angiogenic therapy [16]. This is significant as long-term treatment in younger individuals poses distinct challenges that cannot be sufficiently addressed by assessing shorter-term measures of efficacy alone, such as cumulative toxicity, dose adjustments, interruptions, compliance, quality of life, and combination regimens with other treatments like chemotherapy, surgery, radiation, immunotherapy, and additional targeted therapy [15, 16, 20, 31]. Among bone sarcomas, some clinically significant side effects include pneumothorax, hand-foot syndrome, hypertension, fatigue, lipids abnormalities, and proteinuria, the latter of which may prove especially problematic for patients with pulmonary metastases and who are anticipated to remain on TKI treatment for extended periods [15, 16, 20]. Thus, while anlotinib seems to hold promise as a potentially valuable option for certain children and young adults with bone sarcoma or ASPS, any future studies will need to assess AYA outcomes independently from those of older adults [15, 16, 20, 31].

### Limitations

This study has some limitations. First, substantial heterogeneity was observed due to the inclusion of both randomized and observational studies, varying lines of therapy, and differences in treatment regimens (monotherapy vs. combination therapy). Second, pooling median survival outcomes has inherent methodological limitations and should be interpreted cautiously. Third, the inclusion of multiple sarcoma subtypes, each with distinct biological behavior and treatment response, limits the generalizability of pooled estimates. Finally, all included studies were conducted in China, which may limit external validity to other populations. Some included studies incorporated gastrointestinal stromal tumors (GISTs) within the broader soft tissue sarcoma (STS) population when separate subtype-specific data were not available. Subgroup analysis based on sarcoma subtype was not feasible because the included studies reported heterogeneous and overlapping sarcoma populations, with each cohort comprising different subtype distributions.

### Future directions

Future studies should be larger and multicenter which would help confirm anlotinib’s true benefit and make the results more reliable. Research also needs to look closely at different sarcoma subtypes, since responses vary and some patients seem to benefit more than others. In addition, more trials combining anlotinib with chemotherapy or immunotherapy are needed, as these combinations might enhance its effectiveness. Studies that include biomarkers could help reveal which patients are most likely to respond, while long-term follow-up would show how safe and tolerable the drug really is over time. Future prospective trials should particularly focus on sarcoma subtypes that demonstrated encouraging responses to anti-angiogenic therapy, including alveolar soft part sarcoma (ASPS), leiomyosarcoma, liposarcoma, and osteosarcoma. These subtypes may derive greater benefit from anlotinib because of their angiogenic biological profiles and previously observed sensitivity to tyrosine kinase inhibition.

## Conclusion

This review highlights anlotinib as a hopeful addition to the limited treatment options for advanced sarcomas. Across studies, it consistently prolonged survival and slowed disease progression, often with mild and controllable side effects. These results are encouraging, especially for patients who have already failed standard treatments. However, sarcomas are deeply complex and diverse and that diversity explains why results vary between studies. Future research must be broader, smarter and more personalized. Large multicenter trials and biomarker-based approaches could help reveal which patients truly gain from anlotinib. In summary, anlotinib stands out as a potent, flexible and generally safe option that deserves further exploration. It may not be a cure, but it offers real hope and in a disease as rare and challenging as sarcoma, that is already a meaningful step forward.

## Data Availability

All data generated or analyzed during this study are included in this published article and its supplementary information files.
